# In vivo analysis of a proprietary glass-based adhesive for sternal fixation and stabilization using rabbit and sheep models

**DOI:** 10.1007/s10856-021-06527-5

**Published:** 2021-04-29

**Authors:** Cina Mehrvar, Emily Deignan, Mark Hurtig, Gideon Cohen, Paul Zalzal, Oleg Safir, Adel Alhalawani, Marcello Papini, Mark R. Towler

**Affiliations:** 1 Department of Mechanical and Industrial Engineering, Ryerson University, 350 Victoria St, Toronto, ON M5B 2K3 Canada; 2 Ontario Veterinary College, University of Guelph, 50 Stone Rd E, Guelph, ON N1G 2W1 Canada; 3 Division of Cardiac Surgery, Sunnybrook Hospital, 2075 Bayview Ave, Toronto, ON M4N 3M5 Canada; 4 Oakville Trafalgar Memorial Hospital, 3001 Hospital Gate, Oakville, ON L6M 0L8 Canada; 5 Faculty of Medicine, Department of Surgery, McMaster University, 1200 Main St W, Hamilton, ON L8N 3Z5 Canada; 6 Division of Orthopaedic Surgery, Mount Sinai Hospital, 600 University Ave, Toronto, ON M5G 1X5 Canada; 7 Department of Biomedical Engineering, The University of Akron, 302 E Buchtel Ave, Akron, OH 44325 USA; 8 Li Ka Shing Institute, St. Michael’s Hospital, Bond Street, Toronto, ON M5B1W8 Canada

## Abstract

Wire cerclage remains the standard method of care for sternal fixation, following median sternotomy, despite being beset with complications. An emerging treatment option has been to augment the wires with an adhesive. A patented ionomeric glass (mole fraction: SiO_2_:0.48, ZnO:0.36, CaO:0.12, SrO:0.04) has been used to formulate GPC+, a glass polyalkenoate cement (GPC), by mixing it with poly(acrylic) acid (PAA) and de-ionized water. In a human cadaver study, this material, when applied with wire cerclage, was able to significantly reduce sternal instability. However, the material has yet to be tested in pertinent animal models. Here, after a series of physical and mechanical tests to confirm suitability of the experimental material for implantation, three samples of GPC+ were implanted in either the tibia or femur of three different rabbits, alongside sham defects, for two different time modalities. A further seven samples of GPC+ and one poly(methyl methacrylate) control (PMMA) were implanted in either the tibia or femur of two different sheep. The sheep containing the PMMA was sacrificed at 8 weeks and the other at 16 weeks, to evaluate time dependent biological response. Upon sacrifice, microCT images were acquired and histology slides prepared for analysis. All three GPC+ samples implanted in the rabbit model, for the two time modalities, were characterized by minimal bone resorption along with a mild inflammatory response. Five of the seven GPC+ materials implanted in the sheep model (all three implanted for 8 weeks and two of those implanted for 16 weeks) were associated with mild to moderate immune response, comparable to that observed with PMMA, as well as mild bone resorption. The remaining two GPC + materials (implanted in the sheep model for 16 weeks) exhibited no bone resorption or inflammatory response and appeared to stimulate increased bone density at the implant site. These results suggest that GPC + can be a viable bone adhesive for use in hard tissue applications such as sternal fixation and stabilization.

**Figure:**
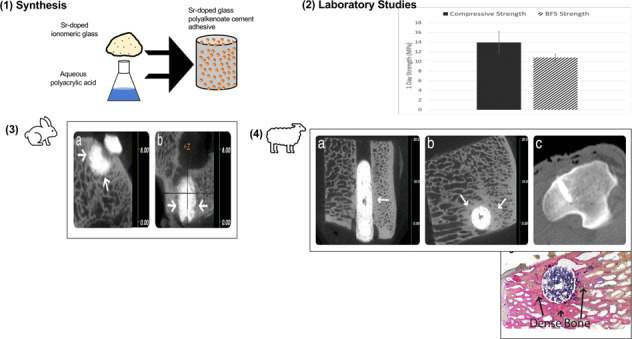
Experiments performed to synthesize & test Sr-doped glass adhesive for sternal fixation. (1) Sr-doped ionomeric glass fired, ground down and mixed with aqueous polyacrylic acid to produce the adhesive. (2) Adhesive characterized and tested by a suite of laboratory-based tests to ensure suitability for implantation. (3) Adhesive implanted into a rabbit model (distal femur, 12 weeks post implantation) where micro-CT images confirmed an excellent bone/cement interface, no evidence of bone resorption and some bone remodelling. (4) Adhesive subsequently implanted into a sheep model; at 16-weeks, a continuous bone—adhesive interface is seen suggesting no bone resorption. There was an increase in the peri-implant radiodensity, suggesting enhanced mineral content of the bone surrounding the GPC+ implant.

Experiments performed to synthesize & test Sr-doped glass adhesive for sternal fixation. (1) Sr-doped ionomeric glass fired, ground down and mixed with aqueous polyacrylic acid to produce the adhesive. (2) Adhesive characterized and tested by a suite of laboratory-based tests to ensure suitability for implantation. (3) Adhesive implanted into a rabbit model (distal femur, 12 weeks post implantation) where micro-CT images confirmed an excellent bone/cement interface, no evidence of bone resorption and some bone remodelling. (4) Adhesive subsequently implanted into a sheep model; at 16-weeks, a continuous bone—adhesive interface is seen suggesting no bone resorption. There was an increase in the peri-implant radiodensity, suggesting enhanced mineral content of the bone surrounding the GPC+ implant.

## Introduction

Median sternotomy, first introduced in 1953 [1], is the standard method of care to gain access to the heart and large vessels with ~700,000 procedures carried out annually in the US alone [2]. Upon completion of the surgical procedure, re-approximation of the sternal halves is required. Wire cerclage, in which stainless steel wires are tightened across the manubrium and in between the rib spaces is the most commonly used method. However, this procedure can result in significant complications [3] including wires cutting through the apposed tissues, infections and postoperative pain; the latter arising from micromotion between the sternal halves, which can be exacerbated by physiological forces such as a cough or a sneeze. As sternal instability develops [4], chronic pain interferes with the quality of life for these patients [5]. Further, sternal dehiscence can occur in up to 5% of patients, exacerbated by risk factors including obesity, diabetes, smoking, and osteoporosis [6]. Such dehiscence is directly linked to deep sternal wound infection and has been associated with a mortality rate of up to 47% [7]. As a result of these complications, alternative sternal fixation and stabilization methods, such as the use of bone cement, are being investigated.

Poly(methyl methacrylate) (PMMA) has been used as a cement in orthopaedic applications since the 1950s, however it is not suitable for sternal applications due to its lack of chemical adherence to bone [8]. In addition, it exhibits dimensional changes during curing [9] and undergoes an exothermic setting reaction that can lead to thermal necrosis [10]. An alternative to PMMA, Kryptonite^™^ bone cement (Doctors Research Group, Southbury, CT, USA), effectively a mixture of castor oil and calcium carbonate, was investigated under an Investigational Device Exemption (IDE) as an adhesive for augmentation of current wire cerclage techniques for sternal fixation [11]. Kryptonite was reported to prevent sternal dehiscence, reduce pain, and improve patient quality of life [12]. However, the Food and Drug Administration issued a recall on Kryptonite due to in-vivo volumetric expansion of up to 49% [13] and a 50% loss of strength at body temperature [14], rendering it unavailable for clinical use [15].

The authors are developing a bone adhesive for sternal fixation augmentation, based on Glass polyalkenoate cement (GPC) chemistry. First developed by Wilson and Kent in 1971 for dental restorative and luting applications [16], GPCs consist of an acid degradable ionomeric glass, poly(acrylic acid) (PAA) and de-ionized (DI) water. Upon mixing, the glass particles undergo a chelation reaction with the aqueous PAA in the presence of DI water [17]. Setting is the result of long PAA chains chelating the cations released from the glass phase, ionically cross-linking them [17]. GPCs chemically adhere to bone [18], exhibit appropriate strength [19], lack volumetric change [20], set without an exothermic reaction [21] and can release therapeutic ions [22]. These properties indicate that GPCs have potential in sternal fixation and stabilization. The material properties of GPCs can be manipulated in a number of ways: increasing the acid concentration [23], the molecular weight of the PAA [24] and the powder:liquid ratio [25], as well as decreasing the particle size of the glass phase:[26] all yield higher cement strengths while lowering working time.

Commercial GPCs all contain aluminum (Al) in the glass phase, which is a neurotoxin [17, 27]. To facilitate the use of these materials for sternal fixation and stabilization, the authors developed and patented (US 7,981,972) an Al-free ionomeric glass (mole fraction: SiO_2_:0.48, ZnO:0.36, CaO:0.12, SrO:0.04), where the Al component is replaced with zinc (Zn) [28]; incorporated for its beneficial effect on bone metabolism [29] as well as its antibacterial and anti-inflammatory properties [30, 31]. Strontium (Sr) is also incorporated in the glass phase to promote pre-osteoblastic cell replication [32] and stimulate bone formation [33]. Zn/Sr-GPCs formulated from this patented glass have been previously reported to significantly reduce sternal instability [34] in a human cadaveric trial.

The first in-vivo investigation of an Al-free GPC was reported in 2016 by Pierlot et al., where four GPC samples were implanted in two rabbits [35]. The glass composition of the GPC investigated (mole fraction: SiO_2_:0.24, GeO_2_:0.24, ZnO:0.36, CaO:0.16) contained germanium (Ge) [35]. The tissue response to the GPC appeared radiologically normal, while the quality of the bone surrounding the GPC was reported to be comparable to that around sham defects [35]. In Pierlot et al.’s study, a collagen membrane (ConFORM Collagen Membrane; ACE Surgical Supply Co., USA) was applied over each defect site; an additional stage in the process which may complicate the surgical operation and influence tissue response [35]. Rabbit models continue to be the most popular option for evaluating possible bone adhesives, used in ~35% of musculoskeletal research studies, mostly because of their ease of handling and relatively low cost [36]. However, extrapolating results from a rabbit to a human is problematic; rabbits have faster bone turnover, as well as significant differences in anatomy and mechanical loading [37] when compared to humans. As a result, rabbits are commonly used to screen implant materials prior to testing them in a larger, more pertinent model.

A sheep model is more suitable than a rabbit model for mimicking human bone, although differences still exist [38–40]. Previous studies have demonstrated new bone formation rates that are three times slower in sheep when compared to rabbits [41]. Sheep bone has a similar remodeling rate to human, but is 1.5–2 times more dense [42]. Furthermore, while adult sheep are in a similar weight range to humans (e.g., A Texel cross ewe weighs 56 ± 7 kg), there are stark biomechanical differences between quadruped and biped models that limit the translation of in-vivo results [39]. Microscopically, sheep have a primary bone structure (osteons < 100 um diameter) while humans have a secondary bone structure (osteons > 100 um) [43].

In this study then, both rabbit and sheep models were used to evaluate the in-vivo response of a GPC (GPC+) formulated from the patented zinc and strontium-containing ionomeric glass (US 7,981,972). Following a series of physical and mechanical tests to confirm clinical suitability of this batch of GPC + , the bioadhesive was implanted into non-critical defects in a bilateral tibiofemoral rabbit model. In the second study, a comparable bilateral tibiofemoral defect in Texel Cross sheep was employed. The desired response in both models was an absence of bone resorption, minimal fibrous encapsulation, and evidence of healthy bone turnover adjacent to the implant.

## Methods

### Glass synthesis and cement formulations

The patented ionomeric glass (SiO_2_:0.48, ZnO:0.36, CaO:0.12, SrO:0.04) was prepared at Ryerson University (Toronto, Canada) using specified mole fractions. Correct amounts of analytical grade reagents (Sigma-Aldrich, Oakville, Canada) were weighed out, thoroughly mixed in a container, and then transferred to a platinum crucible and melted in a furnace (Zircar Hot Spot 110, Florida, New York, USA) at 1480 °C for 1.5 h. The glass melt was water quenched at room temperature (23 ± 1 °C) and the resultant frit dried in an incubator (37 °C) for 24 h. The frit was then ball-milled and sieved to 45–63 µm. The resultant glass powder was then annealed for 12 h at 640 °C, having reached the annealing temperature in 3 h. Following annealing, the glass was furnace cooled to room temperature. The formulation of the resultant GPC + was: 3 g of glass, 0.9 ml of DI water and 0.9 g of PAA (molecular weight, 210 k). The handling and mechanical properties of GPC + have been reported elsewhere [19, 44, 45] but were repeated on this particular GPC + batch for completeness, prior to commencement of animal work.

### Handling properties of GPC+

The working and setting times (*n* = 5) of the GPC + samples were recorded at room temperature (23 °C ± 1°C). The working time was measured, using a stopwatch, from the start of mixing and was considered complete once the material began to exhibit elastic properties.

The setting time was measured in accordance with ISO 9917-1:2007 [46]. A mold with dimensions of 10 × 8 mm was filled to a level surface with mixed cement. A Vicant needle indenter (mass: 400 g, diameter: 1.06 mm) was lowered onto the cement surface and allowed to rest for 5 s. Once the needle was removed, the indent it made was observed. If the indent was visible to the naked eye, this process was repeated on a new part of the surface. Once no indent could be visually observed, the setting time was considered complete.

### Determination of compressive strength

The compressive strengths of the GPC + samples were measured at room temperature (23 ± 1 °C) in accordance with ISO 9917-1:2007 [46]. Cylindrical GPC samples (*n* = 5) of 6 mm height × 4 mm diameter were created by filling plastic tubular molds with mixed cement. The molds were sandwiched between two stainless steel plates protected by a cellulose acetate sheet, and incubated (37 °C) for 1 h. Samples were then removed from the molds, individually placed in 10 ml of DI water and incubated (37 °C) for 1 day.

Compressive loads were applied to the samples using an Instron Universal Testing Machine (Instron Corp., MA, USA) fitted with a ± 10 kN load cell at a crosshead speed of 1 mm/min. The load cell error was calculated at 0.005% at 50 N to 0.04% at 100 N, within which these test samples fracture. The maximum load reached by each sample was recorded. The compressive strength C (Eq. 1) was calculated as,

1 $${\mathrm{C}} = \frac{{4\rho }}{{\pi d^2}}$$

where *ρ* was the maximum load (N) at failure and d the sample diameter (mm).

### Determination of biaxial flexural strength

The biaxial flexural strengths (BFS) of the GPC + samples were evaluated using the method described by Williams et al. [47]. Cement was filled in a tubular mold of 2 mm thickness and 15 mm diameter, sandwiched between two metal plates protected by a cellulose acetate sheet, and placed in an incubator (37 °C) for 1 h. The cement discs were then removed, individually placed in 10 ml of DI water and incubated (37 °C) for 1 day.

The BFS testing was performed using the same Instron Universal Testing Machine (Instron Corp., MA, USA) described in Section *Determination of Compressive Strength* at a crosshead speed of 1 mm/min. The GPC + discs were placed on a 3-ball support, and the load was applied as a point in the middle of the disc. The BFS (Eq. 2) was calculated as,

2 $${\mathrm{BFS}} = \frac{{{\mathrm{F(N)}}}}{{{\mathrm{t}}^2}}\left\{ {0.63\ln \left( {\frac{{\mathrm{a}}}{{\mathrm{t}}}} \right) + 1.156} \right\}$$

where *t* is the thickness of the specimen, a is the radius of the support diameter (3.825 mm), and F is the recorded load at failure

### Animal preparation

All animal procedures were approved by an institutional animal care committee (Ontario Veterinary College, Guelph, Ontario, Canada) operating under the guidelines of the National Council on Animal Care. All procedures were carried out using an aseptic technique under general anesthesia with intra- and postoperative analgesia.

#### Rabbit model

New Zealand White rabbits (*n* = 3, 3.0 ± 0.1 kg) were group housed in a 2 × 5 m pen and conditioned for 1 week prior to the commencement of the study. On day 0, each rabbit was sedated with ketamine (10 mg/kg) and dexmedetomidine (0.01 mg/kg) followed by endotracheal intubation with isoflurane general anesthesia. Hair was removed from the tibial and femoral surgical sites and a skin incision was performed on both hind stifle joints. Blunt dissection was used to clear the subcutaneous tissue. Defects (∅ = 3.5 mm) were made in the left and right distal femur and proximal tibia, allowing for a maximum of four implants per animal. Care was taken to avoid migration into the medullary canal. Sterile saline was used to irrigate the defects, removing osseous drilling particles. One GPC+ cement was implanted in each of the three rabbits (*n* = 3) in accordance to their allocated defect site. In addition, one sham defect was implanted in two of the three rabbits (*n* = 2). One rabbit was sacrificed at 8 weeks post implantation (*n* = 1/3) while the other two rabbits were sacrificed at 12 weeks (*n* = 2/3) for subsequent microCT (45-micron resolution, GE Locus Xplore Scaner) and analysis of undecalcified histology sections.

#### Sheep

Two Texel cross sheep (*n* = 2, 56 ± 7 kg) were group housed in a 3 × 4 m pen and conditioned for 1 week prior to study commencement. On day 0, both sheep were anesthetized with diazepam (0.3 mg/kg) and ketamine (5.0 mg/kg), followed by endotracheal intubation with isoflurane general anesthesia. Wool was removed from the tibial and femoral surgical sites followed by aseptic skin preparation. Upon incising the skin, blunt dissection was used to clear the subcutaneous tissue. Defects (∅ = 6.5 mm) were made in the left and right distal femur and proximal tibia of each animal. Care was taken to avoid migration into the medullary canal and saline irrigation was used to remove osseous drilling particles from the defects. One PMMA cement (Palacos Bone Cement, Heraeus Medican, Hanau, Germany) and 7 GPC+ cements (*n* = 7) were implanted in the two sheep used in this study. One sheep was sacrificed at 8-weeks post implantation, and the other at 16-weeks post implantation, for subsequent microCT and histology analysis. The PMMA control was included as one of the four implants in the sheep sacrificed at 8 weeks.

### Material preparation and implantation

All GPC+ components were sterilized by gamma irradiation at a 25 kGY dose (Model: G.C. 220, 3.6 kGy/hr, University of Toronto, ON, Canada). GPC+ was prepared by placing the PAA component in a glass mixing dish followed by the addition of the DI water component. These were then mixed using a metal spatula until fully homogenous, at which point the ionomeric glass component was added. The cement was mixed for ~30 s until completion, determined by visual confirmation of consistent, homogenous material. The PMMA samples were mixed in a sterile bowl according to the manufacturer’s specifications. Freshly mixed cements were rolled into a cylinder before being lightly packed into the defect by hand. Any excess adhesive around the defect site was removed before closure, where the opening of the defect site was covered with the periosteum, muscle and fascia, subcutaneous tissue and a bandage.

### CT/MicroCT image acquisition

Each implant in the sheep model was imaged at day zero using clinical resolution computed tomography (CT) (Discovery RT, GE Healthcare, ON, Canada) for confirmation of appropriate defect fill. Clinical CTs were not recorded for the rabbit model given the smaller implant size rendering the image resolution too low for effective analysis. Following animal sacrifice of both the rabbits and sheep, microCT imaging was performed on a General Electric Medical Systems Locus Explore Scanner at 45-micron isotropic pixel resolution using an 18 min protocol (Kv = 80, mA = 450). Image slices were reconstructed into 3D images after calibration using a hydroxyapatite (HA) phantom and subsequent image analysis was performed using MicroView (Parallax Innovations, version 2.5.1, Ilderton, Ontario, Canada).

### Histology

After microCT imaging, all implants were processed for undecalcified ground section histology analysis by light microscopy (Langholm Consulting, MA, USA). Extraneous tissue not required for analysis was stripped from the samples upon their extraction. The samples were placed on ice prior to processing and fixed in 70% ethanol. They were then were embedded in methyl methacrylate, and sections in the transverse plane to the cylindrical defect were cut with a thickness of ~15 µm. The resulting sections were stained with Sanderson’s Rapid Bone Stain and Van Gieson’s picrofuchsin. Images were digitally captured (Nikon Eclipse E400, Melville, NY, USA) using standard brightfield techniques at objective magnifications of ×4, ×10, and ×40. The resulting images were analyzed to assess the response of the tissues to the implanted materials. The color of the various specimen components upon Sanderson’s Rapid Bone Stain and Van Gieson’s staining are presented in Table 1.

**Table 1:** Color of various tissues after staining

Cellular feature or tissue type	Color
Bone	Light red to dark red
Cartilage	Purple
Dense collagen / Osteoid	Green
Muscle	Blue
Tendon	Blue
GPCs	Dark blue / black
PMMA	Black

## Results

### Working and setting times

The average (*n* = 5) working and setting times of the GPC+ samples were 4:48 (stdev: 0:07) minutes and 34:06 (stdev: 0:22) min, respectively. The handling properties of this specific batch of GPC+ were in line with those reported for the same material previously [19, 44, 45] and deemed suitable for sternal augmentation by a cardiac surgeon.

### Evaluation of compressive and biaxial flexural strength

**Fig. 1:**
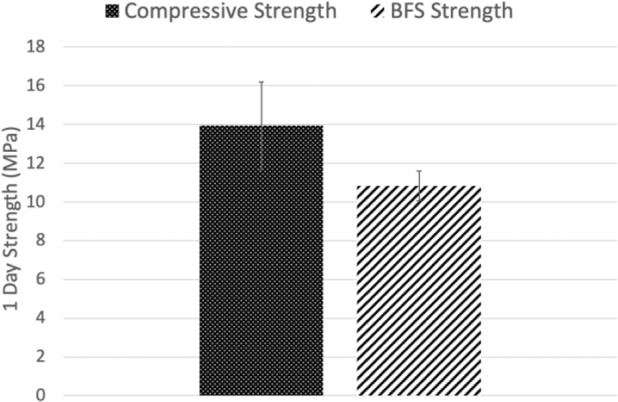
Compressive and Biaxial Flexural Strength of GPC+ at 1 Day (*n* = 5)

The compressive and BFS of GPC+ after 1 day of incubation are presented in Fig. 1. The compressive strength of the cement (~14 MPa) was found to be slightly greater than its biaxial flexural strength (~11 MPa). These properties were in line with those reported for this material previously [19, 44, 45] and deemed suitable for sternotomy augmentation by a cardiac surgeon.

### MicroCT images

#### Rabbit

**Fig. 2:**
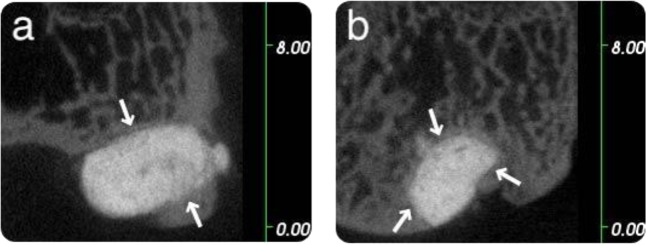
Rabbit microCT images of GPC+ implanted in the left distal femur at 8 weeks. A continuous bone—cement interface is seen as indicated by the arrows (**a**: coronal; **b**: sagittal). Scale is in mm

**Fig. 3:**
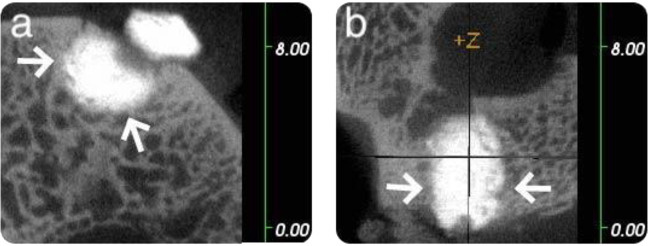
Rabbit microCT images of GPC+ implanted in the left distal femur at 12 weeks. A continuous bone-cement interface is seen as indicated by the arrows (**a**: coronal; **b**: sagittal). Scale is in mm

In the rabbit model, all three microCT images of the GPC+ implants (one obtained at 8 weeks and two obtained at 12 weeks) presented an excellent bone/cement interface, no evidence of bone resorption and some evidence of bone remodelling. Sample microCT images of a GPC+ implant in a rabbit model at 8 weeks are presented in Fig. 2, while microCT images of a GPC+ implant in a rabbit model at 12 weeks are presented in Fig. 3.

There was no apparent difference in tissue response at 8 or 12 weeks (Figs. 2 and 3, respectively). The GPC+ implants contrast strongly with the surrounding bone due to their high radiopacity.

#### Sheep

Two sheep were operated upon. Following surgery, both sheep showed initial moderate lameness in the hind limbs for the first few days, followed by mild lameness at a consistent or intermittent level for the duration of the study. It is relevant to note that this alteration in the natural gait cycle could have spurred abnormal patterns of bone remodelling in limbs with GPC+ implants.

There was a slightly more variable response to the GPC+ implants in sheep. This variability could be attributed to difficulties in respect to drilling into the harder sheep bone, which can be twice as dense as human bone [42], thereby increasing the opportunity for thermal bone injury, which could have prompted more remodelling than was observed in the rabbit model.

**Fig. 4:**
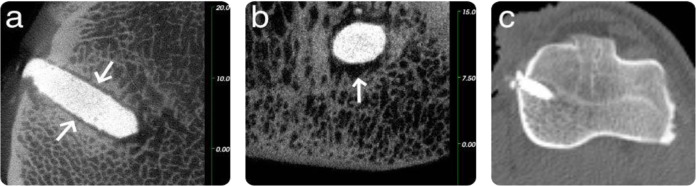
Sheep microCT images of a GPC+ implanted in the left proximal tibia at 8 weeks. Cement appears uniform while a slight lucent border is seen surrounding the implant, as indicated by the arrows. (**a**: coronal, **b**: sagittal, **c**: Day 0 Clinical CT scan). Scale is in mm

Two microCT images of a GPC+ implant acquired at 8 weeks are presented in Fig. 4 alongside a CT scan recorded at the time of implantation (Fig. 4c). The lucent border suggests a mild degree of bone resorption may have occurred, although a lucent border at *t* = 0 may also be present (Fig. 4c), indicating that lucency could be an artefact of the high radiopacity of the GPC+. The GPC+ mass appears uniform suggesting a homogenous mix. CT images of the other GPC+ samples implanted for 8 weeks (not included here, for reasons of succinctness) show comparable responses; a homogeneously mixed cement stable at the implant site, perhaps with evidence of mild bone resorption (although this may be an artefact), minimal fibrous encapsulation and healthy bone turnover adjacent to the implant. Any minor variance in response to the implants may be attributed to implantation and mixing variability; which can occur with experimental materials.

**Fig. 5:**
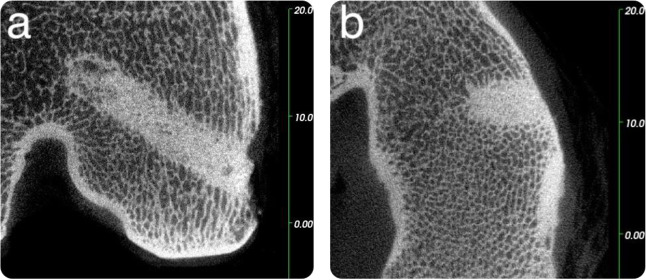
Sheep microCT images of a PMMA implanted in the left distal femur at 8 weeks. A continuous bone—cement interface is observed, while the radiopacity of the PMMA appears less than the GPC+ implants (**a**: coronal, **b**: sagittal) Scale is in mm

The PMMA control (in the sheep sacrificed after 8 weeks) had an intact bone-cement interface but no enhancement of the surrounding bone mineral density (Fig. 5). A continuous bone—cement interface can be seen surrounding the PMMA implant with growth of periosteal bone fully encompassing the cement within the bone. The PMMA implant is notably less radiopaque than the GPCs.

**Fig. 6:**
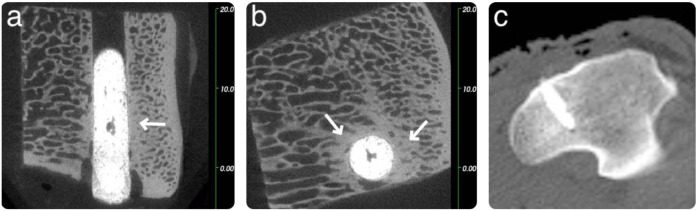
Sheep microCT images of a GPC+ implanted in the left proximal tibia at 16 weeks. A continuous bone—cement interface is observed, areas of increased radiodensity indicated by arrows (**a**: coronal, **b**: sagittal, **c**: Day 0 Clinical CT scan). Scale is in mm

Considering an example of the GPC+ implants at the 16-week time point (Fig. 6), a continuous bone—GPC+ interface can be seen suggesting no bone resorption had occurred. In addition, there was an increase in the peri-implant radiodensity, suggesting enhanced mineral content of the bone surrounding the GPC+ implant. The small area of cortical bone loss at the cortex is likely due to the drilling technique.

The effect of implantation time on the GPC+ samples inserted into a carefully drilled and irrigated host defect can be evaluated by comparing Figs. 4 and 6 (8 weeks and 16 weeks implantation, respectively). Of the GPC+ materials, all three implanted for 8 weeks presented with slight lucency at the implant/bone border. Of the four GPC+ samples implanted for 16 weeks, two presented with slight lucency and two presented a continuous bone-cement interface with no evidence of bone resorption. Lucency may be a result of some minor bone resorption or could be an artefact of the high GPC+ radiopacity.

### Histology

#### Rabbit

**Fig. 7:**
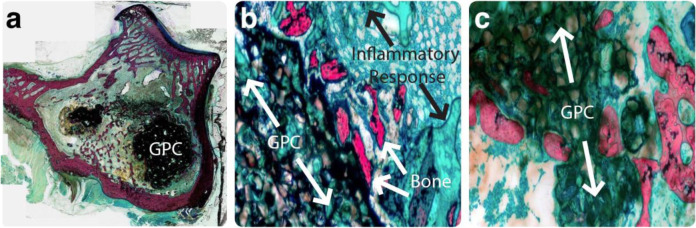
Histological images of a GPC+ implant acquired from a rabbit model at 8 weeks. **a** Slide overview, some bone apposition to cement mass visible. **b** ×10 magnification, a mild inflammatory response is seen adjacent to GPC+. **c** ×10 magnification, little to no cells visible within the bulk of the material

There was no evidence of infection in any of the histological samples processed from the rabbit model. A mild inflammatory response, including an increase in leukocytes and fibrous tissue at the implant interface, was seen around all the implants. Limited bone resorption was seen with around the GPC+, alongside some new bone formation. Few, if any, debris particles were visible in the surrounding tissues. Processed histological images of a representative GPC+ implant are presented in Fig. 7. Bone (red/pink) is seen in direct apposition to the cement (black) and some new bone (osteoid) formation (green) can be seen. While it appears some GPC+ degradation has occurred, this is likely due to activities associated with thinning and processing samples for histological analysis. A limited number of dislodged cement particles are seen surrounding the implant. At higher magnifications (Fig. 7b, c), a mild inflammatory response can be seen adjacent to the cement mass. Few, if any, cells are visible within the bulk of the material and the bone does not appear to be in direct contact with the adhesive. There was no apparent difference in response between implants obtained at the 8 and 12 week timepoints.

#### Sheep

**Fig. 8:**
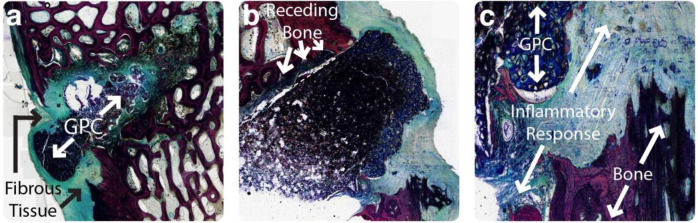
Histological images of a typical GPC+ implant acquired from a sheep model at 8 weeks. **a** Slide overview, some bone found surrounding material; robust fibrous tissue response. **b** ×10 magnification, bone has receded from drill tract and been replaced by fibrous tissue. **c** ×20 magnification, increased inflammatory response visible surrounding implant

**Fig. 9:**
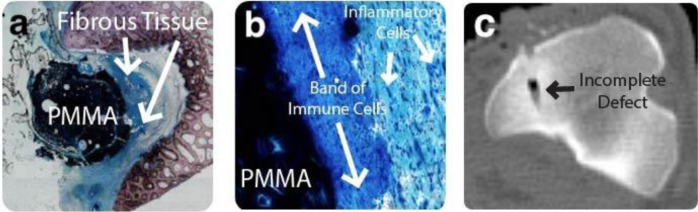
Histological images of a PMMA implant acquired from a sheep model at 8 weeks. **a** Slide overview, unfilled defect with fibrous tissue found between PMMA and bone. **b** ×40 magnification, band of immune cells found directly adjacent to PMMA surface. **c** Postoperative clinical CT scan of PMMA implant with incomplete filling of defect

There was no evidence of infection in any of the samples acquired from the sheep model. A varied histological response was observed around the GPC+ implants. A moderate inflammatory response was seen in the slides of one of the three 8 week samples (Fig. 8) characterized by fibrous tissue and leukocytes in close apposition to the GPC+ surface, where leukocytes increased in density with proximity to the implant (Fig. 8c). In some cases, fragments of dislodged material were present in the surrounding tissues. The inflammatory response observed with respect to these GPC+ implants was comparable to that seen around the PMMA implant (Fig. 9b). Mild bone resorption appeared to occur as a result of increased osteoclast activity (Fig. 8b). GPC+ is visible as a dense mass (dark blue/black) with some evidence of fragmentation. Some bone can be seen both within, and surrounding, the GPC+. There is fibrous tissue response (blue/green) surrounding the implant (Fig. 8a). The bone (red/pink) appears to have retracted from the drill line and been replaced by fibrous tissue and a dense infiltration of immune cells.

A moderate inflammatory response was observed in the histology of the PMMA implanted for 8 weeks (Fig. 9b); comparable to that seen around the GPC+ implants at the same time modality. The PMMA (dark blue/black) appears to have been ejected from the defect, however CT imaging immediately postoperative showed that the cement did not completely fill the defect (Fig. 9c). Furthermore, there appear to be cracks formed on the medial side of the PMMA, potentially releasing wear particles that could contribute to the inflammatory response. Radiologically, the defect did not appear to grow in size through the healing process, suggesting the gap between the bone and PMMA was the result of incomplete filling rather than resorption. Normal osteoblastic activity, as well as an abundance of osteocytes, were observed on the surrounding bone. Upon higher magnification, a band of immune cells were found directly adjacent to the PMMA surface, while individual inflammatory cells are found further away (Fig. 9b).

**Fig. 10:**
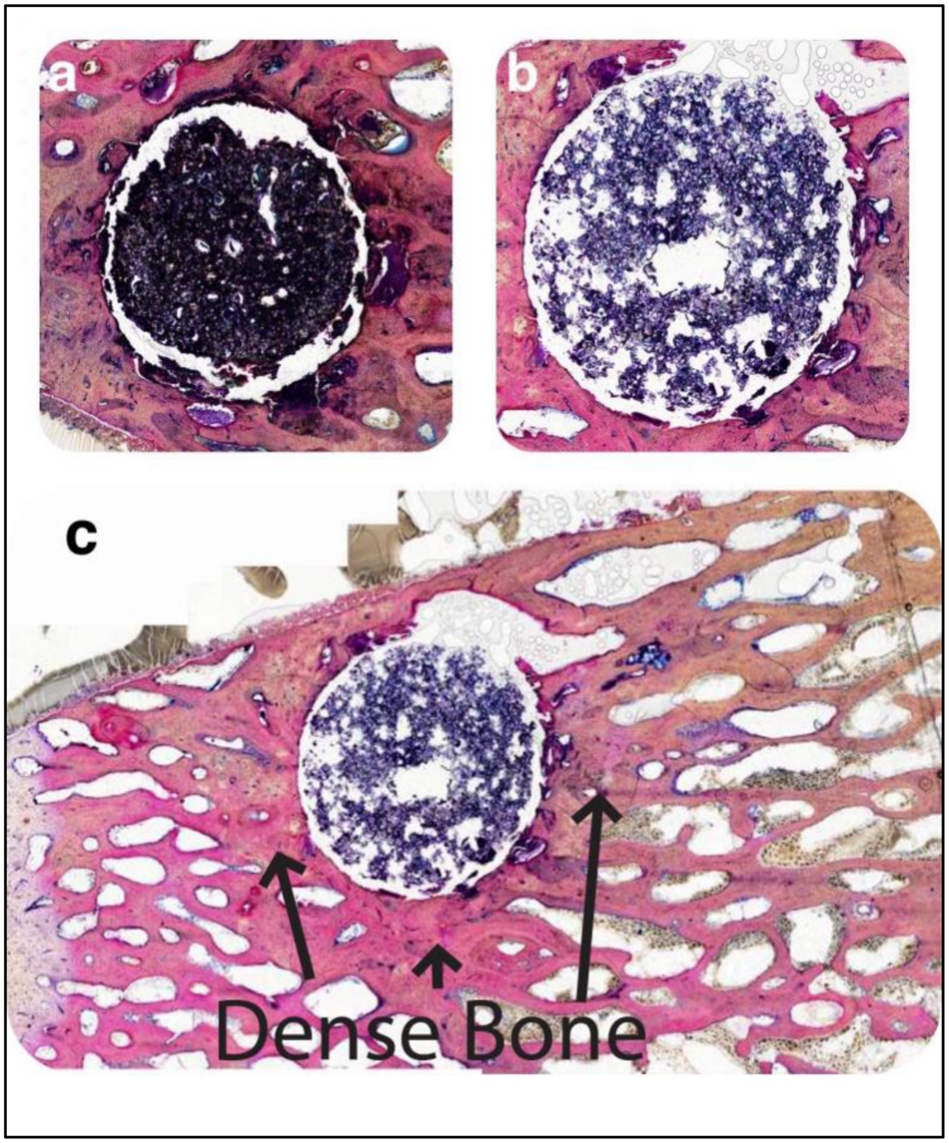
Histological images, taken at different cross-sectional slices, of an exceptional GPC+ implant at ×4 magnification acquired from a sheep model at 16 weeks. **a** Dense layer of bone surrounding implant with no inflammation present. The implant is undergoing some fragmentation and delamination of its surface partially due to shrinkage of during processing. **b** A thinner ground section which makes the material appear more porous, but the surrounding bone is intact and highly compact. **c** Expanded view of the bone around the implant

Considering the histology around the four GPC+ implants that were obtained 16 weeks post implantation, the inflammatory response was characterized as mild to none, with few immune cells evident. Regarding two of those recovered samples (one of which is presented in Fig. 10), minimal fibrous tissue was observed with no bone resorption. In addition, the surrounding bone is more dense than that which is further from the implant (Fig. 10c). This is the ideal response to GPC+ implants, and a result that cannot be achieved by a PMMA implant due to its lack of osteoinductivity. The sclerotic reaction around the cement increased the trabecular thickness and bone volume fraction. The adjacent fatty marrow is normal and there is no fibrous encapsulation or any cell reaction around the cement mass. The observed loss of material between the implant and bone is likely to have occurred during grinding and thinning of the sample, since a continuous bone – cement interface was observed radiologically (Fig. 6).

## Discussion

GPC+, a novel GPC, based on a patented ionomeric strontium zinc silicate glass, is postulated as an adhesive augment for sternal fixation and stabilization. Despite human cadaveric studies confirming its potential in this application, there are no published studies on the in-vivo response to this material. Here, GPC+ was manufactured and evaluated by a suite of physical and mechanical testing modalities prior to its clinical suitability being evaluated through both rabbit and sheep models.

Upon implantation of GPC+, all animals remained healthy throughout the duration of the study, with no evidence of infection. In the rabbit model, all three GPC+ implants were found to have a continuous bone – cement interface with minimal bone resorption coupled with a mild immune response and little to no fibrous encapsulation. These results justified further investigation of such implants in a sheep model.

A mild inflammatory response was seen around the three GPC+ implants in the sheep sacrificed at 8 weeks. This response was mirrored in the PMMA control sample in the same sheep. Some lucency was also observed around the three GPC+ implants under CT imaging, but this may, in part, be due to the high radiopacity of the GPC+ (compared to the PMMA control) giving artifice.

In two of the four GPC+ samples implanted for 16 weeks some lucency was also visible at the interface; in the other two samples, an excellent tissue response was observed, with no bone resorption. There was little evidence of fibrous encapsulation or an inflammatory response around any of the samples implanted for 16 weeks. As can be seen in the CT scans in Fig. 6, representative of the 16 week implants, the bone surrounding the implant may be more dense than the bulk bone (Fig. 10c). Further work is required to determine whether the apparent increase in density is due to the release of ions into the surrounding bone or whether it is a result of the surgical procedure.

This increase in bone density seen in Fig. 10 may be explained by the continuous release of Sr^2+^ by GPC+, providing local administration of Sr^2+^ to the surrounding tissues. There are no examples in the literature of Sr-doped GPCs evaluated in animal trials. However, a review of other Sr-enriched biomaterials indicate that they outperform similar materials free of Sr in bone healing, bioactivity and osteo-integration at the bone cement interface [48]. Further, Sr-enriched biomaterials including CaSi ceramics [49], HA’s [50] and CaP cements [51], have been shown to enhance bone formation. Following implantation of a Sr-enriched calcium phosphate cement, areas of enhanced bone formation were associated with higher Sr^2+^ concentrations. Metals coated with SrO have also been shown to increase bone formation, providing evidence for the positive effect of Sr independent of Ca, Si or Zn release [52]. Despite the positive effects that Sr release is reported to have on bone, excess Sr release can be toxic to the bone, disturbing Ca metabolism and bone mineralization [53]. The tissue response observed around the GPC+ implants in the sheep model indicate no evidence of toxic response, but some beneficial response, to Sr release (Fig. 10).

Bone resorption, as it relates to GPCs, can be caused by a number of factors. An excess of ions can potentially disrupt normal cell function. For example, in-vitro concentrations of Zn^2+^ <400 µm have been reported to cause cell death [54]. Bone resorption can also result from a low pH as osteoclasts are directly stimulated by an acidic environment [55]. PAA is an integral component of a GPC and unreacted PAA may leach during the curing process. In 1998, Blades et al. investigated the biocompatibility of an Al-containing GPC using a rabbit model, reporting poorly mineralized bone and fibrous tissue found directly adjacent to the material [56]. They hypothesized that the poor tissue response was caused by the creation of an acidic environment while the GPC was setting in-vivo. Bone resorption may also be caused by ejection of debris particles into the environment. This can be exacerbated by mechanical loading on an unset cement, which can trigger an immune response that stimulates osteoclastic bone resorption and fibrous encapsulation [57, 58]. Erbe et al., investigating an Al-containing GPC using a rabbit model, reported loose cement particles found in the tissues surrounding the implant along with an influx of macrophages characteristic of an inflammatory response [59]. Early versions of Polyethylene (PE) were associated with poor tissue response as a result of wear particles eliciting an inflammatory reaction [60–62]. PE wear particles were reduced by cross-linking a polymer of ultra-high molecular weight, thereby increasing the cohesive strength and wear resistance of the material [63]. It therefore follows that increasing the cohesive strength of GPCs can be achieved by increasing the molecular weight of the PAA component.

Upon histological analysis of the rabbits in this study (representative images included in Fig. 7), few, if any, debris fragments were found in the surrounding tissues. However, cement fragments could be seen in the surrounding tissue in three sheep GPC+ implants obtained at 8 weeks (representative image, Fig. 8). Macrophages, neutrophils and foreign body giant cells were all seen in vicinity of the dislodged debris fragments. In contrast to these 8 week results, no debris particles were observed surrounding sheep GPC+ obtained at 16 weeks (representative image, Fig. 10).

The moderate inflammatory response coupled with the evidence of bone resorption upon GPC+ implantation into the sheep model was only evident after 8 weeks and not after 16 weeks. Sheep bone turns over much more slowly than both rabbit and human bone [43]. Flautre et al., investigating a Calcium Phosphate bone cement in a sheep, noted that new bone formation would continue for a 24 week period [41]. This would explain why the 16 week sheep GPC+ implants performed better than the 8 week implants, due to the increased time given for healing. As humans have faster bone formation than sheep, a sheep response to an implant may appear worse than a human response at equivalent timepoints as the healing process is less complete. New bone formation in rabbits has been observed to be three times faster than sheep [41], suggesting why the GPC+ rabbit implants performed better than sheep implants at equivalent timepoints.

## Conclusion

GPCs have potential advantages over PMMA in sternal fixation and stabilization as they exhibit greater radiopacity [64], have the potential to enhance bone formation [48] and chemically adhere to bone [65]. GPC+, investigated here, appears suited for sternal fixation: all three GPC+ samples implanted in a rabbit model were characterized by minimal bone resorption along with a mild inflammatory response. Five of seven GPC+ materials implanted in a sheep model (all three implanted for 8 weeks and two implanted for 16 weeks) were associated with mild to moderate immune response, comparable to that observed with PMMA. The remaining two GPC+ samples (implanted in the sheep model for 16 weeks) exhibited no bone resorption or inflammatory response and appeared to stimulate increased bone density at the implant site.

## Future work

To ensure the success of GPC+ in sternal fixation and stabilization, the appropriate ion release concentrations needed to achieve a healthy tissue response and ensure consistent release across samples will be identified. Further, steps should be taken to improve the homogeneity of the material, ideally by the use of a delivery kit that can ensure reproducibility in mixing and application, comparable to that used for the storage, mixing and delivery of commercial PMMA bone cements. In addition, improving the cohesive strength of GPC+ using higher molecular weight PAA should be investigated.

A sheep sternotomy trial where the sternum is dissected and then fixated using GPC+ augmented techniques has been designed and will now be conducted to ensure the effectiveness of the cement in this novel application.
